# Remote Blood Pressure Monitoring in Pregnancies at Risk of Developing Preeclampsia

**DOI:** 10.1007/s11906-025-01332-9

**Published:** 2025-05-28

**Authors:** Theepika Rajkumar, Annemarie Hennessy, Angela Makris

**Affiliations:** 1 School of Medicine, Western Sydney University, Sydney, NSW Australia; 2 Department of Renal Medicine, South Western Sydney Local Health District, Warwick Farm, NSW Australia; 3 University of Sydney, Westmead, NSW Australia; 4 School of Medicine, Western Sydney University, Sydney, Australia; 5 University of New South Wales, Kensington, NSW Australia

**Keywords:** Remote blood pressure monitoring, Preeclampsia, Hypertensive disorders of pregnancy, Self-monitoring of blood pressure, Home blood pressure monitoring

## Abstract

**Purpose of Review:**

This review examines the literature on remote blood pressure monitoring (RBPM) for pregnant women at high risk of hypertensive disorders of pregnancy (HDP).

**Recent Findings:**

Hypertensive disorders of pregnancy are a leading cause of maternal and perinatal morbidity. High risk women often require frequent outpatient review for blood pressure monitoring which can be resource-intensive. RBPM is an organised framework which allows patients to monitor their own blood pressure with clinician guidance, improving healthcare utilisation and potentially saving healthcare costs without worsening maternal and fetal outcomes. Following the COVID-19 pandemic and the growing research interest in mobile health, RBPM has been integrated into international guidelines for managing high-risk pregnancies. Yet there is significant heterogeneity across RBPM frameworks described in the literature, and a lack of clear guidance on the development and implementation of this strategy.

**Summary:**

RBPM offers promising additional surveillance for high-risk pregnant women. However, challenges remain in its safe implementation, including patient selection, technology, costs, and adequate training to ensure accuracy in blood pressure readings.

## Introduction

Hypertensive disorders of pregnancy (HDP), encompassing preeclampsia, gestational hypertension, superimposed preeclampsia, chronic hypertension, white coat hypertension and masked hypertension, are the most common cause of maternal and perinatal morbidity and mortality [1]. Particularly, preeclampsia increases fetal morbidity and mortality by 5–sixfold [2], and the long-term maternal effect of preeclampsia (as well as gestational hypertension) has independently been recognised to increase lifetime risk of chronic hypertension (3.7 times higher), coronary artery disease (2.2 times higher) and stroke (1.8 times higher) [3–5]. Early identification of the risk of preeclampsia, and prevention if possible, is a core tenet of management [3].

The most pertinent risk factors which predispose to preeclampsia are chronic hypertension (RR: 1.38, 95% CI: 1.01–1.87), kidney disease (RR 1.8; 95% CI: 1.5 to 2.1), pre-existing diabetes mellitus (RR: 3.56, 95% CI: 2.54–4.99), a previous hypertensive disorder of pregnancy particularly preeclampsia (RR: 7.19, 95% CI: 5.85–8.83), and autoimmune disease particularly antiphospholipid syndrome (RR: 9.72, 95% CI: 4.34–21.75) [3, 6]. Women with any one of these risk factors are classified as high-risk. Evidence reveals that the rate of HDP in this high-risk population can be as high as 33.8%, with an associated high rate of adverse fetal outcomes (IUGR or pre-term delivery) between 15–17% [7]. Given this, guidelines recommend that women at high risk of preeclampsia:

1. be identified before week 12 of gestation and commence low-dose aspirin (75–150 mg) until delivery [8–11],
2. undertake more frequent blood pressure (BP) measurements than standard antenatal care [6, 12]

Administration of low-dose aspirin to women at high risk has been shown to be beneficial in reducing the risk of preeclampsia [7], yet this benefit is only achieved if aspirin is commenced within a specific time frame, with the overwhelming consensus that aspirin should be commenced between 11–16 weeks’ gestation [13]. With poor aspirin adherence, the incidence of preeclampsia in a high-risk population is as high as 58%. However, in women with good adherence to aspirin, the preeclampsia incidence was reduced to ~ 7% [7]. This rate still remains significantly higher than the global estimated incidence of pre-eclampsia of 4.6% with regional rates varying between 1% and 5.6% [7, 14–16].

Stratification of these high-risk women to models of antenatal care with additional monitoring is the other mainstay of management that is employed to modify outcomes. Early hypertensive treatment can prevent morbidity and potentially mortality, as evidenced in the CHIPS (Control of Hypertension in Pregnancy Study) randomized controlled trial whereby tighter BP control (target diastolic BP 85 mmHg versus target diastolic BP 100 mmHg) was associated with less episodes of severe hypertension, which itself was associated with less adverse maternal and perinatal outcomes on post-hoc analysis. This effect was independent of a diagnosis of preeclampsia [17, 18]. Furthermore, the CHAPS (Chronic Hypertension and Pregnancy) trial added further weight to this, with improved perinatal outcomes in those with chronic hypertension and a BP target of < 140/90 mmHg versus a strategy whereby treatment is withheld until the BP was > 160/105 mmHg [19]. Therefore, improving detection and treatment of raised BP is important [20]. Traditionally, this increased surveillance is undertaken with extra outpatient visits, compared to a lower risk pregnancy, and usually includes review by a high-risk attending midwife, obstetrician, and/or hypertension specialist. This strategy expends considerably more resources at the level of both the patient and healthcare system. Frequent monitoring can be a source of anxiety for these women and their families, is demanding for patients in terms of time, transport costs and work absence, and has significant service implications for healthcare providers [21]. Despite this, elevations in BP may arise between these visits.

Rapidly evolving technology has led to up to 50–60% of hypertensive pregnant women, in some countries, undertaking self-monitoring of their blood pressure [22, 23]. Yet many issues have been identified with self-monitoring without appropriate education and clinician oversight, including lack of knowledge about correct measurement technique, use of an unvalidated device, uncertainty about BP thresholds, and no clear escalation pathways for review [24]. In recent years, there has been an increasing focus on mobile health (mHealth) technologies to make healthcare delivery more efficient. Advances in mobile technology have resulted in clinicians using technology to manage, monitor, and treat a patient’s illness from a distance, and compared to traditional methods of disease surveillance. mHealth has been found to have improved accuracy, reductions in time and cost, and improved data quality [25]. One form of mHealth is remote blood pressure monitoring (RBPM), which refers to an organised framework in which patients monitor and record their own BP using a validated machine. Clinicians review these blood pressure readings and adjust treatment, or provide participants with clear instructions for contacting care teams when blood pressure readings are out of prespecified targets.

In the non-pregnant population, remote BP monitoring has been shown to reduce BP levels [26, 27], improve adherence to antihypertensive medication [28] and reduce rates of GP consultations without an increase in expenditure [29]. Compared with clinic readings, it provides a better estimate of underlying BP and long-term outcomes [30, 31]. Given these benefits in the non-pregnant population, there has been an increasing focus on whether remote monitoring of BP could be utilised in pregnancy with similar advantages, while potentially alleviating the burden of additional outpatient visits, for both high-risk pregnant women and the healthcare system [21]. In a systematic review of clinical practice guidelines for hypertensive disorders of pregnancy, 9 of 15 international guidelines either recognised the utility of, or recommended remote BP monitoring for hypertension control [32]. This included the International Society for the Study of Hypertension in Pregnancy (ISSHP), which has made several recommendations for the inclusion of remote BP monitoring during pregnancy and postnatal period [33]. This review focuses on the evidence for RBPM in pregnant women at increased risk for developing preeclampsia or postpartum women with an established diagnosis of HDP, in line with guideline recommendations.

## Hypertensive Disorder of Pregnancy

Hypertension in pregnancy is defined as a systolic BP (sBP) ≥ 140 mmHg and/or diastolic BP (dBP) ≥ 90 mmHg. These measurements should be confirmed by repeated readings at least 4 h apart [11]. Previously, ISSHP recommended a home blood pressure threshold of > 135/85 mmHg for the diagnosis of hypertension in pregnancy, as it was generally accepted that home BP readings were lower than clinic BP measurements [33]. Yet a large individual patient data meta-analysis showed there is only a mean self-monitoring clinic difference of ≤ 1.2 mmHg sBP throughout pregnancy [34]. Consequently, the diagnostic threshold was revised on the most recent 2021 guidelines, with ISSHP recommending a cut off BP < 140/90 mmHg for clinical and non-clinical settings. Blood pressure can also vary across gestation, yet gestation specific thresholds are not recommended to avoid confusion [35, 36].

Hypertension in pregnancy is classified according to the criteria outlined in Table 1, and once in-clinic BP is found to be elevated (but not where preeclampsia is diagnosed) self-monitoring is advised [9]. The rationale for this is not only early detection and timely treatment of elevated BP, but to also improve detection of white coat or masked hypertension, which are no longer considered to be benign conditions. With white coat hypertension, defined as elevated BP in a clinical setting with normalisation of BP at home, 40% develop gestational hypertension while 8% progress to preeclampsia [37]. Women with white coat hypertension also have an increased risk of delivering a small-for-gestational-age newborn (RR, 2.47; 95% CI, 1.21–5.05) and preterm birth (RR, 2.86; 95% CI, 1.44–5.68) compared with normotensive women [38]. Similarly, masked hypertension, defined as normotension in a clinical setting with elevated BP readings in other settings, is associated with higher rates of preeclampsia with severe features (aOR, 23.35; 95%CI, 14.25–38.26), preterm delivery (aOR, 2.47; 95%CI; 1.55–3.94), caesarean delivery (aOR, 1.58; 95%CI, 1.13–2.23), small for gestational age (aOR, 2.27; 95% CI, 1.31–3.94), and neonatal intensive care unit admission (aOR, 2.20; 95% CI, 1.18–4.09) [39]. Thus, early identification of these entities is a potentially crucial advantage of RBPM.

**Table 1:** Classifications of Hypertensive disorders of pregnancy

Type of hypertensive disorder	Definition
Pre-pregnancy or at < 20 weeks’ gestation	
Chronic hypertension	Hypertension detected pre-pregnancy or before 20 weeks’ gestation • Essential: H ypertension without a known secondary cause • Secondary: Hypertension with a known secondary cause (e.g., renal disease)
White-coat hypertension	sBP ≥ 140 and/or dBP ≥ 90 mmHg when measured in the office or clinic, and normal BP using HBPM or ABPM readings
Masked hypertension	BP that is normal at a clinic/office visit, but ≥ 140/90 mmHg at other times outside the clinic/office
Onset after ≥ 20 weeks gestation	
Gestational hypertension	Hypertension arising de novo at ≥ 20 weeks’ gestation in the absence of proteinuria or other findings suggestive of pre-eclampsia
Pre-eclampsia	
De novo	Pre-eclampsia (de novo) is gestational hypertension accompanied by evidence of end organ dysfunction in one or more of the following at ≥ 20 weeks’ gestation: • Renal: proteinuria (urine protein:creatinine ratio > 30) or AKI (creatinine ≥ 90) • Neurological: eclampsia, altered mental status, blindness, stroke, clonus, severe headaches, or persistent visual scotomata • Haematological: thrombocytopenia (platelet count < 150,000/μ L), DIC, or haemolysis • Liver involvement: elevated transaminases (ALT or AST > 40 IU/L), or right upper quadrant/epigastric abdominal pain • Pulmonary oedema • Uteroplacental dysfunction: placental abruption, fetal growth restriction, abnormal umbilical artery Doppler waveform analysis, or intrauterine fetal death
Superimposed on chronic hypertension	Among women with chronic hypertension, development of new proteinuria, another maternal organ dysfunction(s), or evidence of uteroplacental dysfunction (as above)

## Considerations of Implementing a Remote BP Monitoring Framework

**Fig. 1:**
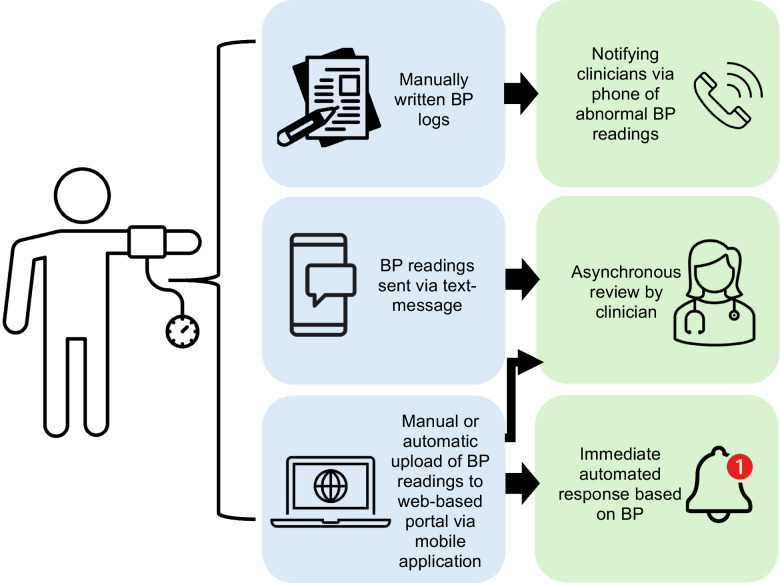
Different frameworks for remote blood pressure monitoring for high risk pregnant women trialed in studies

In a Canadian survey, 60% of pregnant women with a HDP reported to monitoring their own BP at home [22]. Similarly in the UK, around 50% of hypertensive pregnant women monitor their own BP [23]. However, many did not share their readings with their clinicians or use pregnancy-validated BP monitors [23]. This emphasises that though self-monitoring is widespread, there is a need for an evidence-based framework to allow systematic review of home BP readings and institution of appropriate management. The existing literature on RBPM in pregnancy and the postpartum period is heterogenous, due to the variable methods of data flow utilised (Fig. 1), including manually written BP logs [21, 40–42] and patients notifying clinical staff via phone calls of BP out of pre-specified targets [43, 44], BP readings sent via text-message [45–47] as well as manual or automatic upload of BP readings to web-based dashboards via mobile applications [21, 40–42, 48–55]. Within the non-pregnant population, studies have demonstrated erroneous patient reporting of home BP when compared with stored readings within a BP device [56, 57]. Inaccuracies were more likely with uncontrolled BP readings [57], and this may be circumvented by memory-equipped blood pressure devices with transfer to treating teams. Furthermore, the review of home blood pressure readings is an important aspect of an RBPM framework. Within the existing literature, review of digitally transmitted BP readings show that they are predominantly asynchronously reviewed by nursing staff, midwives or doctors [21, 40–42], while some mobile application-based frameworks of RBPM may provide immediate automated responses based on BP readings [48, 51]. However, these studies have not examined the medicolegal implications of reviewing home blood pressure readings, including the review process outside of regular hours, the extent of delay between reading and review, the timely communication of escalation plans to patients, accountability of care, the potential consequences of technology failure and renumeration.

Monitoring schedules are also highly variable across the existing evidence base, and the prespecified BP targets at which participants should seek medical review were also inconsistent, including systolic BP ≥ 155 mmHg or diastolic BP ≥ 100 mmHg [21]; ≥ 140/90 mmHg or ≤ 100/60 mmHg [58] and systolic BP ≥ 150 mmHg or diastolic BP ≥ 100 mmHg [40, 42, 48, 59]

In terms of blood pressure devices, automated oscillometric devices are widely available and straightforward to use in a non-clinical setting, but the physiological changes of pregnancy and preeclampsia may affect accuracy. In preeclampsia, there is altered vascular reactivity, reduced intravascular volume, and reduced vessel compliance, along with interstitial oedema. These changes may affect the amplitude and detection of the oscillometric waveform or alter the validity of the algorithm used to calculate systolic and diastolic BPs [35]. Therefore, devices utilised for a remote BP monitoring strategy needs to be validated for use in both pregnancy and preeclampsia. Validation protocols have been agreed by the British Hypertension Society, the European Society of Hypertension, and the Association for the Advancement of Medical Instrumentation, and strict adherence to an individual protocol is fundamental for accuracy and validity [60]. STRIDEBP provides an up to date list of validated BP devices for use in pregnancy, with 28 devices available on the market currently which are validated for use in this high-risk population (Table 2) [61]. In a systematic review of RBPM in antenatal women at high risk for developing preeclampsia, and postpartum women with a HDP, only 7 out of 18 studies utilised validated BP devices [62]. Guidelines strongly recommend the use of BP devices validated in pregnancy and preeclampsia [9–11, 63, 64] and this is an important consideration in the implementation of RBPM to ensure accuracy of BP readings. This raises further questions about the provision of validated monitors and whether this cost would be incurred by the healthcare system or the patient. During the COVID-19 pandemic, the National Health Service in England provided some BP monitors for those with hypertensive pregnancy, yet many pregnant women were expected to provide their own [65]. In a retrospective survey of clinicians and recently delivered high risk pregnant women that undertook RBPM, around half the women expected RBPM to be free, with funding by the hospital or their health insurance provider, whereas clinicians’ felt that the pregnant women should also personally contribute to the cost of RBPM [66]. The price of a validated blood pressure monitor typically ranges from USD $30 to $180, depending on the retailer and local availability (see Table 2 with approximate pricing in USD). Requiring patients to cover this cost may place an undue burden on individuals from lower socioeconomic backgrounds. This could exacerbate pre-existing challenges often faced by these populations, such as limited access to technology, poor internet connectivity, and lower digital literacy. However, RBPM also offers a potential benefit: it may help reduce disparities in healthcare access for those living in regional and rural areas, who are similarly affected by socioeconomic disadvantage.

**Table 2:** Validated devices in pregnancy and preeclampsia with approximate USD pricing

Andon iHealth Track ($29.99)	Omron M400 Comfort (HEM-7155-D) ($79.99)
Avita BPM636 ($39.99) Grandway G.LAB MD41 A0 ($59.99) Ideal Life Blood Pressure Manager ($99.99) Microlife BP 3BTO-A ($33.00) Microlife WatchBP Home ($79.50) Microlife WatchBP Home A ($79.50) Microlife WatchBP Home A BT ($115.29) Microlife WatchBP Home S ($69.10) Omron Evolv (HEM-7600 T-E) ($129.00) Omron M3 Comfort (HEM-7155-ALRU) ($70) Omron M3 Comfort(HEM-7155-E) ($53.89) Omron M4 Intelli IT (HEM-7155 T-ALRU) ($70) Omron M4 Intelli IT(HEM-7155 T-EBK) ($89.99)	Omron M400 Intelli IT (HEM-7155 T-D) ($70) Omron M500 Intelli IT (HEM-7361 T-D) ($99.99) Omron M6 Comfort (HEM-7360-E) ($129.99) Omron M6 Comfort AFib(HEM-7380-E) ($128.69) Omron M7 Inteli IT(HEM-7361 T-EBK) ($149.00) Omron M7 Intelli IT (HEM-7361 T-ALRU) ($147.00) Omron M7 Intelli IT Afib (HEM-7380 T1-EBK) ($149.99) Omron X3 Comfort(HEM-7155-EO) ($69.99) Omron X4 Smart (HEM-7155 T-ESL) ($129.99) Omron X7 Smart Afib (HEM-7380 T1-EOSL) ($150.00) Omron X7 Smart(HEM-7361 T-ESL) ($179.99) Withings BPM Connect ($129.95) Withings BPM Connect Pro ($199.95)

Additionally, patient selection is crucial for the successful implementation of an RBPM framework. Remote monitoring is unlikely to be effectively adopted by individuals who do not speak the native language, have low literacy, or lack access to consistent network connectivity—factors that align with the exclusion criteria used in RBPM trials. Additionally, individuals who declined participation due to factors such as high anxiety, which could hinder their compliance, should also be considered.

Other important facilitators to implementation of RBPM includes adequate training of midwives, clinicians and patients. Healthcare staff need dedicated training to the technical aspects of RBPM and troubleshooting common problems [66]. Patients’ need education on the technique of self-monitoring, as blood pressure measurements are affected by maternal position, inappropriate cuff size, conversation, caffeine, smoking and an irregular heart rate [35]. Information technology support for technical issues and operating software upgrades, incorporation into existing electronic medical records, security of data transfer and storage, and access to reliable technology (Table 3).

**Table 3:** Key aspects of consideration for implementing an RBPM framework

Healthcare staff	- Adequate training and education - Medicolegal ramifications - Renumeration - Accountability of care
Patients	- Patient selection (low literacy, non-native language, anxiety) - Adequate training and education
Financial considerations	- Cost of blood pressure devices - Cost of RBPM framework - Funding source
Information technology	- Technical support - Incorporation into existing electronic medical records - Security of data transfer and storage - Access to reliable technology and network connectivity
RBPM framework	- Blood pressure devices validated in pregnancy and preeclampsia - Accurate data flow - Blood pressure thresholds for escalation

## Maternal and Fetal Outcomes

Early RBPM studies and a 2020 systematic review consolidating the predominantly observational data that existed at the time, found no increase in adverse maternal, fetal and neonatal outcomes [21, 41, 48, 49, 67–69]. Subsequently, the COVID-19 pandemic instigated major change to clinical practice. With the need to limit social movement, women with HDP or at high-risk of developing HDP were priority groups to undertake home monitoring of blood pressure [70]. As a result, there have been several large randomised controlled trials evaluating remote blood pressure monitoring strategies in the proceeding years. Three large randomised controlled trials, involving 3445 participants, were conducted to evaluate antenatal remote blood pressure monitoring in high-risk pregnant women [40, 42, 48]. These were all conducted by the same authors in the United Kingdom, as part of the Blood Pressure Monitoring in High Risk Pregnancy to Improve the Detection and Monitoring of Hypertension (BUMP) trials, and they found that, compared to conventional care, RBPM did not lead to significantly earlier detection of hypertension [71] or better control of blood pressure [72]. Importantly, they found no difference in adverse maternal and fetal outcomes between RBPM and conventional care [48, 71, 72]. This is consistent with the findings of a recent systematic review of RBPM in pregnant women at high risk of developing preeclampsia, which included eighteen studies with 28,094 patients, revealing that when compared with clinic BP monitoring, there was no significant difference in the likelihood of caesarean section deliveries or induction of labor due to hypertension, a composite maternal outcome, growth restriction, neonatal intensive care unit admissions, gestational age at delivery and a composite fetal outcome [62]. Yet the applicability of these findings to different healthcare systems remains less well studied.

## Health Care Utilisation

Almost universally, the existing literature reports less outpatient antenatal visits and day assessment unit/outpatient antenatal testing unit attendances when utilising RBMP compared with conventional clinic monitoring [21, 41, 43, 68, 73], though this was often inherent to the study design of the remote monitoring group. In contrast, the large, linked BUMP trials reported no difference in day assessment unit/outpatient antenatal testing unit attendances or outpatient clinic visits between the RBPM and conventional care arms [74]. Regarding antenatal hospitalisations, findings are mixed [21, 49, 68, 73, 75]. A recent systematic review and meta-analysis revealed that antenatal RBPM was associated with a reduced number of outpatient visits (standard median difference −0.51; 95% CI, −0.71 − −0.31; I^2^ = 0%; 2 studies, 402 women), and a reduced number of antenatal admissions for hypertension (OR, 0.20; 95% CI, 0.09 − 0.44; I^2^ = 0%; 2 studies, 303 women), with no difference in antenatal admissions for all causes [62].

## End-user Perceptions of RBPM

Early pilot studies exploring the use of remote BP monitoring showed that it was acceptable and feasible to pregnant women. Recruitment to studies ranged from 71–84% [48, 73], while retention of participants was around 80% [59]. Persistence with remote monitoring was also high, ranging between 66–93% [48, 49, 59, 76]. Qualitative surveys have shown that remote monitoring was not only considered acceptable, but may be favourable to conventional clinic monitoring of BP. Most women found RBPM easy to use [43, 75, 77, 78], felt more involved in antenatal care [76], found it assisted with healthy pregnancy-related behaviours [78], preferred it to conventional care as it was less anxiety provoking [77, 79] and would recommend remote monitoring to other pregnant women [43, 76]. Most participants did not have concerns about the privacy of their health data when entered into mHealth applications for RBPM [66]. A minority of women reported that self-monitoring led to uncertainty and unwanted responsibility [24]. One study found that the most common reason to decline participation in RBPM was due to health-related anxiety [80].

Clinicians have reported good adoption and improved engagement in care by patients, and did not perceive a change in their workload [79]. However, uncertainty remains around home-clinic differences and which readings should inform clinical decisions, with some clinicians disregarding home readings if discrepancies exist [34, 80]. Other important factors that clinicians felt were essential for implementation of RBPM included the need to receive additional training on the technical handling of the devices, as well as appropriate counselling and consent of patients [66]. Overall, clinicians supported RBPM and would recommend it to their patients and colleagues [66].

## Cost-effectiveness

The evidence for the cost-effectiveness of RBPM is currently varied. A case control trial from the UK estimated that an antenatal remote BP monitoring strategy for high-risk pregnant women could lead to a mean saving per week of £200.69 for the UK healthcare system [67]. In the cost analyses from the linked BUMP1 and BUMP2 randomised controlled trials in the UK, there was no statistically significant difference in the mean total costs between the RBPM intervention and usual care arm, though RBPM had slightly higher total costs overall [74]. In BUMP 1, which recruited women at high-risk for developing preeclampsia, the mean difference in total costs was £151 [95% CI, − £633 to £936]. For the BUMP 2 chronic hypertension cohort, the mean difference was £323 [95%CI − £2904 to £3549] and for the gestational hypertension cohort the mean difference was £41 (95% CI, − £2486 to £2567) [74]. A Belgian cost analysis from the retrospective Pregnancy Remote Monitoring (PREMOM) study, evaluating an antenatal RBPM framework for women with a diagnosis of HDP, revealed an overall reduction of €740.38 (14.89% of total costs) per person for the healthcare system for remote monitoring, when compared with conventional care [81]. The greatest difference in costs between RBPM and conventional care was in the group that delivered before 34 weeks’ gestation (a reduction of 50.52% [€9,125.17]), with the cost savings predominantly in neonatal care [82]. Importantly, some of these studies did not consider the costs associated with organising a comprehensive RBPM framework, including the price of blood pressure devices, the healthcare staff who supervised the data, and the technical support [67, 82].

## Postpartum RBPM

Unlike the initial observational studies which evaluated antenatal RBPM, the early postpartum studies assessing RBPM were large prospective case–control studies and randomised controlled trials [46, 51, 52]. This may relate to postpartum women being a more convenient population to study. They have had a definitive diagnosis of hypertensive disorder of pregnancy, are recruited following delivery, and only require remote monitoring for a short, defined period, usually 6 weeks to 3 months postpartum. Without the risk to fetal outcomes, this also makes the postpartum period a safer time to conduct a trial. These studies similarly revealed good feasibility and acceptability of RBPM when undertaken in the postpartum period. Recruitment was lower than in the antenatal data, ranging from 47–59% [51, 83], however retention rates were high, ranging from 83–95% [83, 84]. A meta-analysis of randomised controlled trial data revealed that when compared with conventional clinic monitoring, women undertaking postpartum RBPM were more likely to have a blood pressure ascertainment or health professional follow-up within 10 days postpartum [OR, 5.60; 95% CI, 1.52 − 20.69; I^2^ = 81%; 3 studies, 494 women], as recommended by the American College of Obstetrics and Gynecology for postpartum women with an established HDP diagnosis [62]. There is moderate strength of evidence that RBPM may even reduce racial disparities in postpartum follow-up [85]. Interestingly, they were also more likely to have unscheduled hypertension-related visits to the outpatient clinic or triage [OR, 2.82; 95% CI, 1.29 − 6.18; I^2^ = 0%; 2 studies, 403 women] [62].Though a number of studies reported fewer hypertension-related hospital admissions when undertaking remote monitoring, compared to clinic monitoring [46, 52, 83], the systematic review revealed no difference in postpartum readmissions for hypertension or outpatient initiation of antihypertensive agents [62].

## Conclusions

Self-monitoring of BP in women at high risk of preeclampsia is increasingly commonplace. Remote blood pressure monitoring is an organised framework in which self-monitoring is undertaken with clinician oversight, to provide additional surveillance to routine antenatal care. There has been increasing interest in utilising RBPM in pregnancy and the postpartum for high-risk women, and this is reflected by the growing evidence base in recent years. The existing literature reveals improved healthcare utilisation with reduced antenatal outpatient visits and antenatal admissions for hypertension, which potentially translates to cost-savings for the healthcare system without an increase in adverse perinatal and maternal outcomes. In the postpartum period, RBPM may lead to more unscheduled presentations for hypertension, but this did not lead to an increase in postpartum hypertension admissions. It may however improve postpartum follow-up. Following the COVID-19 pandemic, RBPM has been included in national and international guidelines in the management of high-risk pregnant women, yet some key questions remain for researchers and policy-makers. There is significant heterogeneity in RBPM frameworks, and safe implementation of an RBPM strategy requires careful consideration of patient selection, technological and financial aspects, as well as adequate training of clinicians’ and patients. This is critical to ensure accuracy and reliability of BP readings in pregnancy, a time where physiology evolves quickly and stakes are high.

## Data Availability

No datasets were generated or analysed during the current study.
